# Lifestyle Interventions to Reduce Diabetes and Cardiovascular Disease Risk Among Children

**DOI:** 10.1007/s11892-014-0557-2

**Published:** 2014-10-26

**Authors:** Dorothy J. Van Buren, Tiffany L. Tibbs

**Affiliations:** 1 Department of Psychiatry, Washington University School of Medicine, Campus Box 8134, 660 South Euclid, St. Louis, MO 63110 USA; 2 School of Advanced Studies and College of Social Sciences, University of Phoenix, 3157 E. Elwood St., Phoenix, AZ 85034 USA

**Keywords:** Cardiovascular, Diabetes, Risk factors, Lifestyle interventions, Pediatric

## Abstract

Diseases once associated with older adulthood, type 2 diabetes and cardiovascular disease, are increasingly diagnosed in children and adolescents. Interventions designed to assist adults in modifying dietary and physical activity habits have been shown to help prevent the development of type 2 diabetes and cardiovascular disease in adults. Given the unfortunate rise in both of these diseases in pediatric populations, it is increasingly important to begin prevention efforts in childhood or prenatally. There is strong empirical support for utilizing lifestyle interventions to prevent these diseases in adults; it is not clear whether the same holds true for pediatric populations. The present review examines lifestyle management efforts to prevent type 2 diabetes and cardiovascular disease in children across socioecological levels. Recommendations are made for expanding the traditional focus of lifestyle interventions from dietary and physical activity behaviors to target additional risks for these diseases such as smoking and depression in youth.

## Introduction

Heart disease is the leading cause of death among adults in the USA [1], and sadly, the markers for adult cardiovascular disease (CVD) are often evident in childhood [2]. In addition to increasing concerns about CVD risk factors in children, type 2 diabetes (T2D), once considered a disease of older adults, is also on the rise in pediatric populations [3–5]. Many adults with T2D die from CVD [6], and risk factors for CVD appear to emerge early in youth with T2D [7]. Furthermore, obesity is a risk factor for the development of both T2D and CVD [8, 9]. Unfortunately, many of the lifestyle behaviors that accompany these risk factors in adults, such as physical inactivity, poor eating habits, and smoking, have their roots in childhood and adolescence [10], and risk factors for both CVD and T2D that can be tracked from childhood into adulthood increase the likelihood of adverse health outcomes in adulthood [11•]. Therefore, it is hoped that early screening for these risk factors in children and adolescents and intervention to address these unhealthy lifestyle behaviors may help prevent the development of these diseases in later years. Randomized clinical trials have provided evidence that lifestyle interventions can prevent diabetes [12–15, 6] and reduce CVD risk in adults [15, 16], but it is not clear whether the same holds true for pediatric populations. This review surveys recent studies designed to impact lifestyle behaviors in young people to determine their ability to decrease risk for developing CVD and/or T2D in the future.

## Risk Factors Associated with the Development of CVD and T2D

An array of cardiometabolic risk factors in children and adolescents, alone and in combination, is associated with the development of CVD and T2D. Early signs of risk for CVD in children, such as high triglycerides, have been associated with higher adult CVD events when these elevations continue into adulthood [11•]. When elevations in triglycerides and high blood pressure during childhood persist into adulthood, these individuals are also more likely to have T2D as adults [11•]. Various markers of inflammation such as C-reactive protein (CRP), fibrinogen, and interleukin-6 have also been identified as important risk factors for CVD in children [17, 18]. In addition, there is a strong evidence that metabolic syndrome in childhood is associated with metabolic syndrome in adults, subclinical atherosclerosis, and T2D, independent of other predictors [19]. Diabetes risk in adults is particularly high in individuals who were obese as adolescents [20]. Persistent overweight throughout childhood, adolescence, and adulthood is associated with a 12-fold increase in the odds of developing T2D as an adult [21]. It has also been suggested that impairments in glucose tolerance in obese children may more rapidly progress to T2D in this age group compared to adults due to the tendency for obese children to gain excessive amounts of weight [22•, 23]. For these reasons, obesity prevention or weight loss in overweight children are often the focus of lifestyle interventions in the hopes that weight regulation in youth will reduce their risk for developing CVD and T2D. In addition, lifestyle interventions have been shown to reduce cardiometabolic risk markers such as inflammation and insulin resistance in children [17, 18, 24, 25]. Pediatric lifestyle interventions typically target the dietary and physical activity habits of at-risk youth as described in more detail below.

### Dietary Behaviors

Several dietary behaviors have been associated with obesity and CVD in children and adolescents such as an increase in the intake of foods with saturated/trans fatty acids, cholesterol, salt, and sugar [26, 27]; thus, these are common intervention targets. In their meta-analytic review of studies of the effects of sugar-sweetened beverages (SSBs) on risk for T2D, Malik and colleagues found a strong connection between higher intake of these beverages and metabolic syndrome and T2D in adults [28]. SSB intake is also associated with CVD risk in adults and obesity and risk of T2D in children [29]. Decreasing SSB intake in adolescents has been shown to have a positive impact on weight [30]. For these reasons, lifestyle management programs designed to prevent CVD, or to treat obesity/overweight in children to prevent the development of T2D and CVD, often set as treatment goals decreased consumption of energy-dense foods and SSBs [2]. Although increasing fruit and vegetable intake is also a frequent treatment target for lifestyle interventions [2], in their review of the literature, Ledoux and colleagues failed to find a clear relationship between increasing fruit and vegetable intake and improvements in weight status [31].

### Physical Activity Behaviors

Changes in diet combined with increases in physical activity promote weight loss which in turn improves insulin resistance and hypertension [2]. However, increased physical activity may positively impact risk factors independent of dietary behaviors. Walking to school was associated with improvements in cardiometabolic risk factors in Portuguese students [32]. When the investigators of this cross-sectional study controlled for dietary fat intake and other forms of moderate to vigorous physical activity, they found that walking to/from school had a positive effect on waist circumference. Strength training may also help in lowering CVD risk and in protecting against insulin resistance [33]. Physical activity and cardiorespiratory fitness in youth are both correlated with insulin sensitivity independent of adiposity, especially when physical activity is at higher intensities [34]. Fedewa and colleagues [35] conducted a meta-analytic review to determine the effect of exercise training on predictors of T2D in children and adolescents. They found small to moderate effect sizes for exercise training on fasting insulin providing support for the inclusion of physical activity targets in lifestyle management programs to prevent T2D in youth.

In order to decrease the risk of CVD and T2D in pediatric populations, lifestyle interventions to promote the adoption of healthier eating and physical activity behaviors have been implemented across a variety of socioecological levels—individual/family or home, school or community, and at the national or policy level [36–39, 40•]. Representative examples of lifestyle interventions applied across these various socioenvironmental levels are described in more detail in the following sections.

## Levels and Focus of Lifestyle Change Interventions

### Individual-/Family-/Home-Based Interventions

Most lifestyle interventions at the individual or family level target both dietary and physical activity behavior changes such as Balagopal et al. [17] who tested the effect of a combined lifestyle intervention on insulin resistance and markers of inflammation in obese adolescents (Table 1). Their participants were able to reverse obesity-related markers of inflammation after 3 months of participation in the lifestyle change intervention despite negligible changes in body weight. However, there were significant decreases in body fat mass and insulin resistance. The study of Kalarchian et al. of a multicomponent family-based intervention with severely obese children is encouraging because participants showed improvements in cardiometabolic factors that persisted into follow-up even though differences in weight between the intervention and the usual care participants did not persist [41]. Some lifestyle change interventions have focused more exclusively on manipulating the macronutrient composition of diet without inclusion of an exercise component. For example, Garnett et al. [24] examined medication plus either a high-carbohydrate, low-fat diet or a moderate-carbohydrate, increased protein diet among overweight or obese 10–17-year-olds with prediabetes and/or insulin resistance [24]. After 6 months of dietary treatment plus metformin, both insulin sensitivity and body mass index (BMI) 95th percentiles were improved compared to baseline values for both intervention groups with no significant differences between them. On the other hand, Ebbeling et al. [30] conducted a small study of obese adolescents that found that a low glycemic load diet (through home provision of water and diet beverages to displace consumption of SSBs) was superior to a more traditional low-fat diet for weight loss and improving insulin resistance.

**Table 1:** Interventions to prevent T2D and/or CVD in youth

	Participants	Study design	Outcomes	Components	Results
Individual-/family-based studies					
Balagopal et al. [17]	21 adolescents: 6 lean and 15 obese matched for age and pubertal status	RCT; 3-month lifestyle intervention or usual care; 6 lean participants baseline measures only	CRP, IL-6, fibrinogen, BMI, weight change, LDL-C, HDL-C, HOMA-IR	D, PA, F	Intervention group showed significant changes or improvements in BMI and weight, %BF, HOMA-IR, LDL-C to HDL-C, CRP, fibrinogen, and IL-6 compared to usual care. Intervention group maintained weight, while the control group gained weight
Ebbeling et al. [30]	16 obese adolescents	RCT; 6-month dietary intervention (reduced glycemic load (GL) versus reduced fat) with 6-month follow-up	BMI, fat mass, HOMA-IR	D	Reduced-GL group showed greater improvements in adiposity and less of an increase in HOMA-IR compared to the reduced fat group
Garnett et al. [24]	98 overweight/obese youth, 10–17 years, with prediabetes or clinical features of insulin resistance	RCT; 6-month intervention; metformin plus high-carbohydrate diet or metformin plus moderate-carbohydrate, increased protein diet; physical activity component added to both groups after 3 months of dietary intervention	Insulin sensitivity, fasting insulin to glucose ratio, BMI, TG, HDL-C, LDL-C, BP	D, PA, F	After 6 months of intervention, both insulin sensitivity and BMI 95th percentiles were significantly lower than at baseline for both groups. No significant differences observed between the two groups on any outcome
Kalarchian et al. [41]	192 obese children, 8–12 years	RCT; 12-month intervention family-based weight loss intervention or usual care	%OW, BMI, WC, %BF, BP	D, PA, F	After 6 months of treatment, there were significant changes in %OW for participants in the family-based treatment that were not maintained at follow-up (12 months). Significant improvements in medical risk factors (BP, WC, %BF) were maintained at follow-up by participants in the family-based treatment condition
Patrick et al. [42]	101 overweight or obese youth, 12–16 years at risk for T2DM	RCT (Pace-Internet for Diabetes Prevention Intervention—PACEi-DP); compared three 1-year obesity treatments to usual care—website only, website plus monthly group sessions and follow-up calls, or website and SMS	BMI, %BF, measures of adiposity, physical activity, sedentary behavior, diet	D, PA, F	The website-only arm had a greater decrease in sedentary behavior than the usual care arm, but there were no treatment effects for the main outcomes of BMI, adiposity, physical activity, or diet at 12 months
School-/community-based studies					
Grey et al. [53]	198 7th graders at risk for T2DM	4 schools randomized to general education (GE) plus coping skills training (CST) intervention and 2 schools to GE alone. All received 8 classes of education CST schools got 5 additional classes in CST and 9 months of telephone health coaching	BMI, %BF, WC, HOMA-IR, fasting insulin, 2-h insulin, fasting OGTT, 2-OGTT, HbA1c, TC, TG, HDL-C, LDL-C	D, PA	2-OGTT levels were significantly reduced, and HDL-C levels were increased somewhat (but did not reach significance) in the CST group compared to the GE group. WC and %BF decreased significantly across both groups from baseline to 4 months, and the effect was sustained at 12 months. Improvements in HOMA, fasting insulin, and 2-h insulin levels across both groups over 12 months
HEALTHY Study Group [52]	4,603 students, assessed at the beginning of 6th grade and at the end of 8th grade	Cluster randomized design, multicomponent school-based intervention (21 schools) or assessment only control (21 schools)	BMI, WC, and fasting glucose and insulin levels	D, PA	The intervention was associated with a significant decrease in the prevalence of obesity in the subgroup of students who were overweight or obese at the beginning of the study and approached significance in the full sample. Significantly greater reductions in: BMI *z* score, the percentage of students with WC >90th percentile, and the mean insulin level in the intervention schools versus the control schools
Paul et al. [54]	862 enrolled and 535 completed the 12-week ENERGIZE! Program for children aged 6–18 years with metabolic syndrome, prediabetes, or T2DM	Community-based program—3 days per week over 12 weeks, to educate families about healthy eating, physical activity, and behavior change. Maintenance phase includes reevaluation every 6 months for 2 years. Assessments at baseline, 6, 12 18, and 24 months	BMI, BP, fasting lipid levels, and fasting blood glucose, fitness evaluations including tests of flexibility, endurance, and muscular strength, and health behavior questionnaires	D, PA, F	At 6 months, significant reductions in mean BMI percentile, TC, LDL-C, TG, systolic and diastolic BP, compared to baseline. Significant reduction in fasting glucose at 6 months for those with impaired fasting glucose at baseline; significant reductions in metabolic syndrome at 6 months compared to baseline. Improvements in BMI, glucose level, lipid levels, and BP sustained at 12 months for participants who continued to participate in the program
Savoye et al. [22]	75 obese youth with prediabetes 10 to 16 years	Parallel group, RCT comparing effects of the Bright Bodies (BB) Program with standard clinical care (CC); program participants met twice per week for 6 months	OGTT, BMI, BMI *z* scores, BF, BP, HOMA-IR, HbA1c, TC, HDL-C, LDL-C, and TG, ALT	D, PA, F	After 6 months, BB had significantly decreased 2-h glucose and lowered BMI *z* scores due to body weight maintenance close to baseline values; the CC group continued to gain weight. The BB group had greater improvements in systolic BP, fasting TG, reduced total BF, marked improvements in insulin sensitivity, and statistically and clinically significant improvements in glucose tolerance
Vivian et al. [43]	46 children, 10–16 years at risk for T2DM, completed the study	Intervention was conducted at 2 neighborhood community centers. Youth were randomized to a control group (*n* = 20) or intervention group (*n* = 26) for the 1-year intervention	BMI *z* score, WC, hemoglobin A1c, fasting glucose, fasting insulin, TC, TG, LDL-C, HDL-C	D, PA, F	Although intervention group had more clinically favorable outcomes compared to the control group, no statistically significant differences were found between the intervention (*n* = 26) and control groups (*n* = 20)

*RCT* randomized controlled trial, *BMI* body mass index, *CRP* C-reactive protein, *IL-6* interleukin-6, *BF* body fat, *HDL-C* high-density lipoprotein cholesterol, *LDL-C* low-density lipoprotein cholesterol levels, *T2DM* type 2 diabetes mellitus, *SMS* short message service, *BP* blood pressure, *HOMA*-IR homeostasis model assessment of insulin resistance, *TC* total cholesterol, *TG* triglycerides, *WC* waist circumference, *D* dietary, *PA* physical activity, *F* family involvement

Children are not the only individuals targeted by lifestyle change interventions since parental weight loss has been shown to predict weight loss in overweight children when parents have been encouraged to lose weight along with their children [44]. In fact, Boutelle et al. [45] found that for every 1 BMI unit reduction in parents, their children experienced a 0.255 reduction in BMI units. Therefore, encouraging weight loss in parents who are overweight should be included in any family- or home-based obesity prevention program for children.

While it is apparent that the involvement and support of parents in behavior change programs is critical for the success for youth at risk for diabetes and CVD, the long-term impact of family-based lifestyle intervention efforts toward prevention of risk factors for T2D and CVD remains to be seen. Some experts in the obesity field would argue that efforts to prevent the devastating health effects of obesity start too late if they begin in childhood [46]. Improving the health of prospective parents may be an important focus in diabetes prevention efforts [38]. The PREMA study (PREdiction of Metabolic syndrome in Adolescence) found that low birth weight, small head circumference, and parental history of overweight or obesity may identify children at risk of developing metabolic syndrome in adolescence [19]. Therefore, prenatal interventions with prospective parents may be useful to reduce future diabetes risk in children.

Unfortunately, it may be difficult to create changes at the individual level since our current environment is so obesigenic [47]. Even with targeted early prevention programs, overcoming these larger societal issues is difficult [48]. Family-based interventions are important, as the analysis of Johnson et al. of the community-based pediatric obesity prevention program, “Be Active Eat Well,” suggests. They found that the home environment has more influence on zBMI than the school environment [40•], but other studies have shown that community-based programs to prevent obesity in children benefit from the inclusion of dietary and physical activity components that are implemented within the schools as well [37, 49•]. Examples of some of these studies are provided in the next section.

### School-/Community-Based Interventions

Recent reviews by Sobol-Goldberg et al. [50•] and Wang et al. [51•] suggest that school-based obesity prevention interventions can be effective in reducing BMI among children, particularly for those programs with more comprehensive content, involving parental support, and duration longer than 1 year. Wang et al. [51•] concluded that there is a strong evidence that school-based studies of physical activity, that include a home component, improve obesity outcomes. Two of the three studies reviewed by Wang et al. [51•] focused on reducing sedentary activity, which may have contributed to the positive results. In addition, combined interventions of diet and physical activity interventions in schools that included home and community involvement were also effective [51•].

In addition to weight outcomes, a variety of school-based studies show promising results specifically related to other diabetes, cardiovascular, or metabolic risk factors. The HEALTHY study was a comprehensive school-based obesity prevention program which resulted in reductions in the prevalence of obesity among those students who were overweight or obese at the beginning of the study. This program also led to a significantly greater reduction in the intervention schools in BMI *z* score, percentage of students with waist circumference in the 90th percentile, and mean insulin levels [52]. Grey et al. [53] also tested a multicomponent lifestyle intervention delivered to inner city, minority youth at risk for T2D within the school. They investigated the addition of coping skills training (CST) and health coaching to see if these would improve outcomes by addressing participants’ barriers to incorporating lifestyle changes. Schools were randomized to either the CST intervention (four schools) or the general education (GE) intervention (two schools). All seventh graders in the schools received the same nutrition and activity educational component (eight classes), but the CST schools received an additional five classes in CST and the youth identified as at-risk for T2D received 9 months of telephone health coaching. The participants from the schools randomized to the CST intervention evidenced improvements in some key markers of metabolic risk of T2DM at the end of the intervention (Table 1).

### Coordinating Community Interventions Across Social-Ecological Levels

Other community-based programs have involved the coordination of community screening events and clinic-based treatments in an effort to reduce T2D and CVD risk in young people [54, 22•]. Paul et al. [54] describe the ENERGIZE! program which was delivered through a community collaboration of local physicians, fitness organizations, and the Wake Forest Medical Center. The program was designed to identify prediabetes or metabolic syndrome in overweight children and to provide them and their families with access to an intensive community-based lifestyle program designed to prevent T2D and other obesity-associated comorbidities through the adoption of a healthier lifestyle. The ENERGIZE! program reduced prediabetes and metabolic syndrome in the at-risk, overweight children who completed the program. Savoye and colleagues [22•] evaluated the effects of the Bright Bodies (BB) Healthy Lifestyle Program on 2-h oral glucose tolerance test (OGTT) results in comparison with adolescents receiving standard of care. The intervention group attended exercise and nutrition/behavior modification classes over the course of 6 months. The BB program significantly decreased 2-h glucose in children at high risk for diabetes after 6 months. In addition, the intervention group lowered BMI *z* scores by maintaining weight close to baseline values, while the control group continued to gain weight. The BB group also had greater improvements in systolic blood pressure, fasting triglycerides, reduced total body fat, improvements in insulin sensitivity, and statistically and clinically significant improvements in glucose tolerance. An ongoing clinical trial, the Stanford GOALS program, exemplifies this trend toward ever more comprehensive obesity prevention efforts [55]. This 3-year, randomized clinical trial is designed to test a coordinated family, school, and community lifestyle intervention compared to an active health education placebo condition on anthropometric as well as cardiometabolic outcomes such as lipids, HbA1c, and CRP.

### Public Health Initiatives and Interventions

**Fig. 1:**
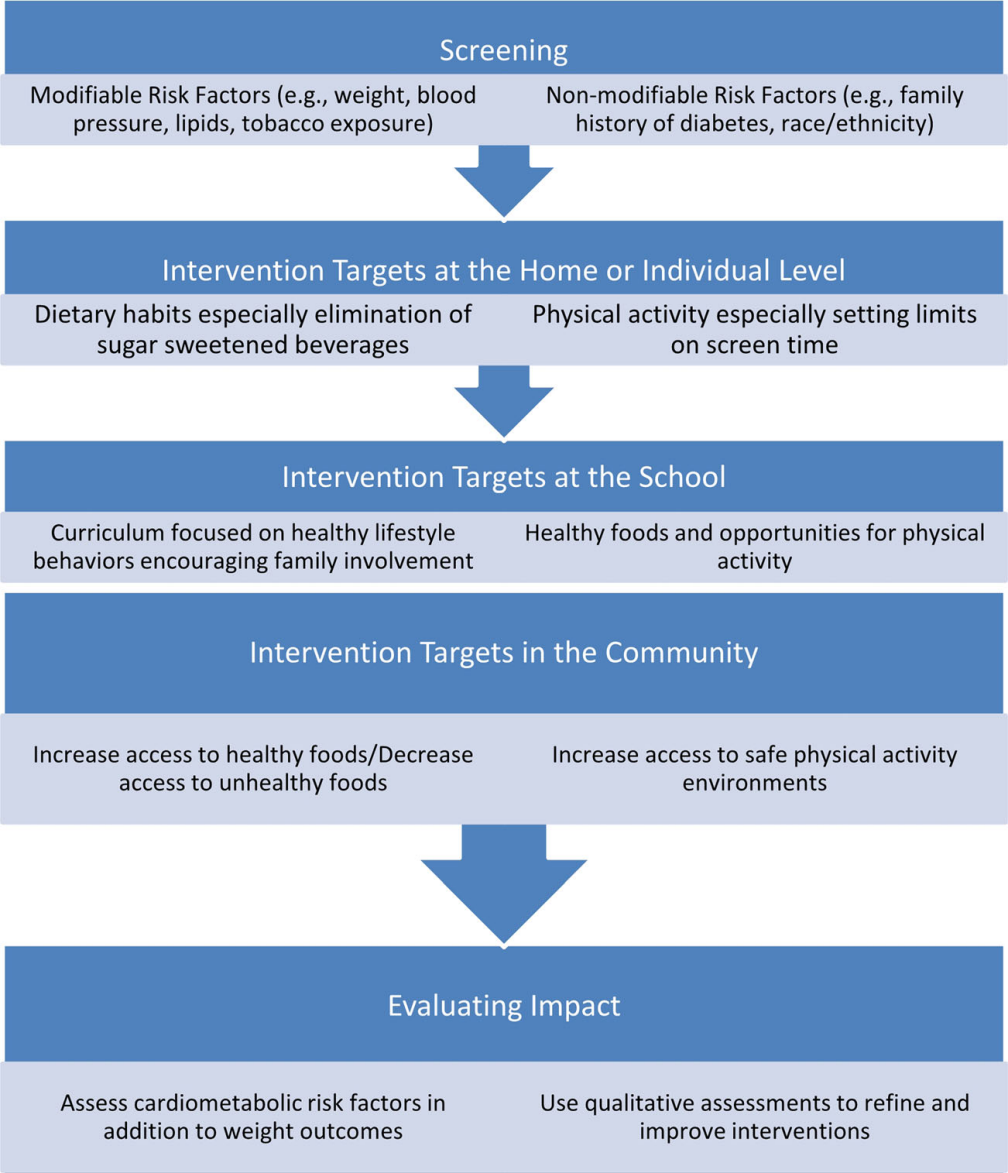
Components of comprehensive interventions to reduce CVD and T2D in youth

Several public health initiatives have been launched at the national and international levels in an effort to decrease children’s CVD risk factors. The World Heart Federation has created an advocacy program for youth called the Youth for Health (Y4H) campaign in which children are encouraged to mentor and educate their peers on the importance of preventing CVD risk factors in their lives [10]. The American Heart Association and the Clinton Foundation sponsor The Alliance for a Healthier Generation which works across several sociocultural levels, families, schools, corporations, and health-care providers, to prevent childhood obesity which is associated with increased risk for CVD (https://www.healthiergeneration.org/). The First Lady’s signature program, “Let’s Move!,” seeks to improve children’s health and decrease CVD risk factors by increasing children’s physical activity, improving the nutritional quality of their school lunches, and increasing families’ access to healthy food and activity (http://www.letsmove.gov/). The Centers for Disease Control and Prevention’s Steps program (also known as the Steps to a Healthier US program) is another initiative targeting the prevention of chronic diseases such as T2D and CVD in youth [56]. The biomedical results from a state-level study, the Carolina Abecedarian Project (ABC), have recently been analyzed. This early intervention initiative targeted disadvantaged youth between ages 0 and 5 years and resulted in significantly lower prevalence of risk factors for CVD and metabolic diseases when the participants were assessed in their mid-30s. The ABC project has demonstrated the persistence of early intervention benefits into adulthood [57•], and more such longitudinal studies are needed to determine whether lifestyle-induced changes targeting cardiometabolic risk factors in childhood persist over the long term. An illustration of the necessary steps and the levels of intervention to consider when designing a community or population-level lifestyle program for reducing pediatric CVD and T2D risk by preventing obesity is provided in Fig. 1. These steps are elaborated upon in the 2014 toolkit titled, Childhood Obesity Prevention Strategies for Rural Communities [58•], providing guidance for the design and implementation of broad-based childhood obesity prevention programs.

## Additional Targets for Lifestyle Interventions to Reduce Children’s Risk of T2D and CVD

Although lifestyle interventions aimed at reducing the risk of T2D and CVD have traditionally focused on dietary and physical activity behaviors, there is a growing body of evidence identifying other modifiable risk behaviors that should be included as targets in lifestyle interventions to prevent noncommunicable diseases such as T2D and CVD [59]. Smoking, sleep, and mental health such as depression are a few examples of the concerns that warrant attention in the design of future risk reduction efforts. While smoking has long been associated with CVD risk, it has been implicated as a risk factor for T2D as well [60, 61]. Smoking that begins at an early age (age 16) has been found to be associated with increased risk for T2D for men [62]. Therefore, CVD and T2D prevention efforts with youth would benefit from including smoking cessation treatment components [63].

There is evidence in adults that there is a U-shaped relationship between sleep duration and diabetes risk since both long and short sleep durations have been found to be associated with increased risk for type 2 diabetes [64, 65]. However, sleep disturbance as a risk factor for CVD has yet to be demonstrated [64]. When sleep duration was examined in adults at risk for T2D who had participated in a lifestyle intervention targeting dietary and physical activity changes, positive changes in self-reported sleep patterns accompanied improvements in nutrition, physical activity levels, and weight loss in this adult population [66]. Additional research into the role of sleep disturbance and risk for T2D and CVD in youth is warranted since Matthews and colleagues [67] found a relationship between short sleep duration on insulin resistance in youth but not for long sleep duration [67].

Depression has also been identified as a risk factor for the development of T2D [68, 69] and CVD [70] in adults. Recent research studies with youth have also found a relationship between depressive symptoms or mood and risk factors for diabetes [71•] and CVD [72–74]. Additional research is needed to more fully investigate the possible interplay between these various lifestyle behaviors, as they have the potential to impact eating, exercise, weight, and overall health outcomes.

## Conclusions

Various professional organizations have proposed guidelines for early identification and prevention of risk for the development of T2D and CVD in children [75, 76], but reducing the risk factors associated with these diseases in our youth is a daunting goal. However, there is some evidence from the National Health and Nutrition Examination Survey (NHANES) that indicates that childhood obesity rates in the USA may have leveled [77] in the past several years, with some decreases in preschool-aged groups, although the authors indicate that the results should be interpreted with caution. There may be small, but hopeful changes in the overall prevalence of childhood obesity as a result of current obesity intervention and prevention efforts.

There are many possible reasons for the decrease in obesity rates, ranging from population changes in breast-feeding to local, state, and federal initiatives and policy changes involving education and healthier food and activity offerings in communities. However, lifestyle interventions to prevent or to intervene with the cardiometabolic markers associated with the development of T2D or CVD may need to begin at ever younger ages especially in the case of obesity prevention. To prevent obesity in children, it has been suggested that lifestyle interventions should focus on those children at high risk for obesity: children with BMIs in the 85th–95th percentiles, who have a family history of obesity in one or both parents, who experienced early onset of increasing weight during their first year of life, who have excessive weight increases during adolescence (particularly in African American girls), who previously engaged in high levels of activity but who have become inactive, and/or those youth who are inactive in general during adolescence [1]. Therefore, intensive public health efforts are needed that involve a variety of different stakeholders to target change at personal, environmental, and socioeconomic levels [78]. Such efforts need to be sustainable, economically feasible, and culturally acceptable so that active-living policies can be effectively implemented across multiple domains [79].

Prevention of T2D and CVD may be classified as primary or secondary due to the length of time these diseases “incubate” [80], and obesity prevention efforts can focus on primary prevention of obesity, secondary prevention including the prevention of weight regain following weight loss, or limiting weight gain in obese people who have not been successful at losing weight [81]. Lifestyle interventions to reduce obesity in adolescents in order to prevent T2D or CVD may be targeting individuals too late to be considered preventative since the early signs of both of these diseases are often evident once a youth has gained excessive weight [80]. Therefore, prevention of childhood overweight may be an even more appropriate target for preventing T2D and CVD than weight loss initiatives [36, 19], particularly since obesity is very challenging to treat once it is established [36]. For these reasons, organizations such as the National Institute for Health and Care Excellence guidelines [82] recommend a focus on all people achieving and maintaining a healthy weight in order to have the most substantial impact on the prevalence and financial costs of T2D. The National Heart, Lung, and Blood Institute’s (NHLBI) Expert Panel’s Guidelines for Cardiovascular Health and Risk Reduction in Children and Adolescents also notes the importance of maintaining a healthy weight in childhood to prevent the development of CVD in adulthood [1].

Lifestyle interventions have primarily focused on changing dietary and physical activity behaviors, but interventions designed to prevent T2D and CVD may improve prevention outcomes by targeting additional health behaviors such as sleep habits [67], stress management or mental health treatment [83], and smoking [2]. Also, our intervention designs have lagged behind in embracing technology to help effect change. Much of our research investigating lifestyle interventions for young people and their families have relied upon traditional education and behavior change methodology (e.g., paper and pencil self-monitoring of eating and exercise behaviors, hard or soft-bound educational materials and handouts, in-person coaching, and teaching in clinics or other community settings). However, the youth today are familiar with and perhaps more comfortable using computerized technology to learn new things and to track behaviors. Technology such as telephones, computers, and web-based interventions has begun to be utilized in lifestyle interventions with good results [84, 85], and more research is needed to determine in what ways the evermore sophisticated personal communication and computing devices as well as social media can be used to impact health behaviors to reduce the risk of T2D and CVD in youth.

Evaluating the cost effectiveness of lifestyle interventions to prevent CVD and T2D is another area for increased study. Saha et al. [86•] note the challenges in comparing the cost effectiveness of various approaches to prevention of chronic diseases such as T2D and CVD. Despite these challenges, lifestyle interventions to prevent disease so far appear to be attractive from a cost-effectiveness perspective particularly when applied within the school or community settings [86•].
